# Prototypes of Hate and Expectations of the Model Victim

**DOI:** 10.1177/08862605241229720

**Published:** 2024-02-20

**Authors:** Caroline Erentzen, Regina A. Schuller

**Affiliations:** 1 Toronto Metropolitan University, ON, Canada; 2 York University, Toronto, ON, Canada

**Keywords:** hate crimes, community violence, violence against, GLBT

## Abstract

This research explored the content of hate crime prototypes in a North American context, with particular attention to how such prototypes might influence blame attributions. In Study 1a, participants were recruited from a blended sample of universities (*n* = 110) and community members (*n* = 102) and asked to report their thoughts about typical hate crime offenses, victims, and offenders. These open-ended responses were coded, and common themes were identified. In Study 1b, a new group of participants (*n* = 290) were presented with these themes and asked to rate each for their characteristics of hate crimes. Studies 1a and 1b confirmed the presence of a clear prototype of hate crimes, such that (a) perpetrators were believed to be lower status White men with clear expressions of bias, (b) hate crime offenses were believed to be acts of interpersonal violence accompanied by slurs or verbal abuse, and (c) hate crime victims were thought to be members of a marginalized group who remain passive during the offense. Study 2 explored the consequences of victim prototypes on assessments of victim blame. Participants (*n* = 296) were recruited from York University and presented with a case vignette that varied the prototypicality of a victim of hate, depicting him as either Black or White and either passive, verbally responsive, or physically confrontational in the context of an assault. Participants showed greatest sympathy for the Black victim who passively ignored verbal harassment but increasingly assigned blame when the Black victim spoke or reacted physically. When the victim was White, participants showed little variation in their assessment of blame as a function of the victim’s behavior. These results suggest that Black victims are subjected to greater behavioral scrutiny than White victims and that sympathy for victims of hate may be contingent on their passivity in the face of harassment.

Hate crimes reflect the extreme boundaries of intergroup prejudice, in which an offender has decided to violate criminal laws to inflict harm on a victim due to their membership in a social group (Iganski, 2001). Compared to similar offenses that are non-hate-based, hate crimes have consistently been shown to cause more physical, psychological, and emotional harm to the victim (Iganski & Lagou, 2014; Paterson et al., 2018). In addition, hate crimes may be thought of as having multiple layers of harm. As Iganski (2001) observes, at the most proximal level is harm to the victim, but there are various outward ripple effects of harm that include members of the targeted community and other marginalized social groups. In this way, hate crimes are ‘message crimes’ that communicate threats and lack of safety to entire communities, reflecting an “*in terrorem*” effect (Perry & Alvi, 2012). For these reasons, hate crimes are treated legally as more serious offenses, meriting sentencing enhancements. In other words, hate crimes do “hurt more” and require harsher penalties (Iganski, 2001, p. 626).

Within a North American context, the Federal Bureau of Investigation recorded 11,288 police-reported hate crimes in 2022 (Federal Bureau of Investigation, 2023). Nearly 60% of these were motivated by racial bias (most often targeting Black victims), 17% by religious bias (most often targeting Jewish victims), and 17% by sexual orientation bias (most often targeting gay men), with the remaining offenses distributed across other identities such as gender identity and disability. Similar results are observed in Canada, with 3,576 hate crimes reported to police in 2022, 54% of which are racially-motivated (primarily Black victims), 21% religion-motivated (primarily Jewish victims), and 14% targeting sexual orientation (primarily gay men; Statistics Canada, 2023). Note that these records may obscure the intersectional nature of hate crime victimization, as research suggests that victims typically feel that they have been targeted for multiple identities, although police may record only one motivation (see Erentzen & Schuller, 2020).

Although definitions and rules vary across jurisdictions, a hate crime is generally any criminal offense that is motivated by bias or antagonism toward the victim’s real or perceived membership in a social group (e.g., Chakraborti & Garland, 2012; Perry, 2001). The Canadian Criminal Code (1985) has limited specific hate crime provisions but does create a blanket sentencing enhancement to any offense where there is evidence of bias motivation (*Canadian Criminal Code, s. 718.2(a)(i)*). A similar approach is adopted federally in the United States via the Federal Bureau of Investigation’s definition of hate crime and the Matthew Shephard and James Byrd Jr. Hate Crimes Prevention Act (2009). There is, however, considerable variation between individual states regarding which groups receive protection under hate crime laws. Some states recognize police and firefighters as protected classes, several recognize homelessness or political affiliation, whereas 17 states do not yet recognize sexual orientation, and 38 do not recognize gender identity as protected classes (Bills & Vaughn, 2023).

## Observers’ Responses to Hate Crime

Where there is evidence that an offense is hate-motivated, observers typically react with contempt toward the perpetrator and recommend harsher penalties (Cramer et al., 2013, 2014; Rayburn et al., 2003; Saucier et al., 2006). Cramer et al. (2010) found that participants were more willing to recommend the death sentence when there was evidence that the crime was motivated by hatred. Similarly, Plumm and Terrence (2013) discovered that merely labeling an assault as a “hate crime” led to higher ratings of guilt, higher ratings of the victim’s mental stability, and lower ratings of the reasonableness of the attacker’s actions. These effects seemed to hold regardless of the specific minority group targeted. For example, Rayburn et al. (2003) found that crimes targeting a minority victim (i.e., Black, gay, or Jewish) versus a White victim were considered more typical of a hate crime and resulted in higher perpetrator blame and lower victim blame. Moreover, hate crimes that fit the general pattern of a White perpetrator and a racialized minority victim are more likely to result in charges being laid by police (Lyons & Roberts, 2014), suggesting that both lay observers and professional actors may be influenced by the relative identities of the perpetrator and victim.

A small group of studies have attempted to explore lay beliefs about hate crimes. An early attempt by Craig and Waldo (1996) revealed that participants imagined hate crimes to involve an act of violence committed by a White assailant against a minority group member. Saucier et al. (2010) found that cross-racial crimes were deemed more typical of hate than intra-racial crimes, but particularly where the offense was committed by a White male. Lyons (2008) similarly found that cross-race offenses were considered more typical of hate crimes and that Black men and gay men were considered the most representative hate crime victims. Similarly, Saucier et al. (2006) found that Black, East Asian, Latino, Jewish, and gay victims were deemed more typical of hate crime victims relative to White victims.

These findings suggest that there may be a prototype of hate crime in North America, with important implications for observer reactions to hate crime offenses. Identifying those elements that might be associated with hate crime is crucial for understanding how offenses are recognized as hate-motivated and how observers react to such offenses. The present research explored the potential existence of hate crime prototypes, including a detailed and systematic study of their nature and content and the possible effects that these prototypes might have on legal decision making.

## Ideal Victims

The concept of an ideal victim was discussed in earlier work by Christie (2018 [1986]), who described an ideal victim as one who is weaker than the offender, who is carrying out a respectable activity, is not blameworthy in some way (e.g., through dress and location), is unknown to the offender, and who is powerful enough to make a complaint but not so powerful that they threaten existing social systems. The offender, on the other hand, is “big and bad” and more powerful than the victim (Christie, 2018 [1986]). This ideal victim description is a more general approach, although some authors have extended it to the context of hate crime. For example, Mason-Bish (2018) notes that ideal victim concepts have created categories of legitimate targets of hate crime, identifying “very particular groups of victims as worthy of the enhanced protection that such laws afford” (p. 45).

Some groups are regularly targeted for violence or intimidation due to their identities but are not protected by hate crime legislation (e.g., goths, sex workers, and homeless persons; see Chakraborti & Garland, 2012; Garland, 2010). Mason (2014) notes that protected groups are those thought to merit compassion, show sufficient vulnerability without being too remote or strange, and not challenge society’s moral beliefs. More recently, Godzisz and Mazurczak (2023) applied Christie’s ideal victim model to observe reactions to anti-LGBT hate crimes with a large European sample. Overall, greater empathy was shown for heterosexual victims relative to gay or lesbian victims, and empathy was reduced as the victim was engaged in supposedly blameworthy behavior, such as being intoxicated or standing near a gay bar or pride parade. It is unclear how Christie (2018 [1986]) developed the ideal victim model or whether it is indeed applicable to hate crime victimization. This model is also relatively unverified and untested despite having a commonsense appeal. It is also unclear whether all ideal victims will receive empathy versus blaming and how various elements of that model interact.

## The Role of Prototypes

A useful approach to understanding and systematically studying expectations of hate crimes may be found in schematic processing research. According to this approach, people rely on prototypes of general categories/concepts in order to process information quickly and efficiently. A prototype is a mental exemplar of a category, the standard against which other similar objects might be compared (Rosch, 1975, 1978). If an object is similar enough to a prototype, we may consider that the object appropriately belongs in that category. If it does not appear similar enough to the prototype, it will not belong in that category. This categorization process applies not only to concrete objects (e.g., animals and furniture) but also to abstract concepts (e.g., social groups and types of crime). The role of lay prototypes within a legal context was explored by Smith (1991, 1993), who found that jurors indeed hold prototypic mental representations of criminal offense categories. Accordingly, jurors compare the facts of a case against their own notion of what constitutes a certain type of crime, regardless of the actual legal requirements for that crime (Kahan, 2015; Smith, 1991, 1993). As a result, jurors are more likely to convict a defendant if the crime fits their prototype, even if a less prototypical crime better satisfies the legal requirements.

Lay prototypes have been studied in several legal contexts, including legal insanity (Kahan, 2015; Louden & Skeem, 2007), sexual assault (Klippenstine & Schuller, 2012), and intimate partner violence (Russell & Melillo, 2006; Waserhaley et al., 2017). For example, mock jurors typically expect “true” rape victims to be emotionally distraught, to have reported to police immediately, to abstain from alcohol consumption, and to have had no prior sexual relationship with the perpetrator (e.g., Klippenstine & Schuller, 2012). Waserhaley et al. (2017) found that gender stereotypes of femininity impacted legal decision making in the context of intimate partner violence involving a lesbian couple (i.e., more masculine female defendants were deemed more responsible for the offense than feminine defendants). Might a similar process occur with hate crimes as well? That is, where a hate crime perpetrator or victim does not match existing lay prototypes, it is possible that observers will be less likely to recognize the offense as hate-based.

## Overview of Present Research

To determine whether there are indeed prototypes of hate crime, the present research employed a systematic study of potential hate crime prototypes following traditional prototype methods (e.g., Louden & Skeem, 2007; Smith, 1991, 1993). In Study 1a, participants were invited to provide their thoughts about a typical hate crime victim, a typical hate crime perpetrator, and a typical hate crime offense in an open-ended format. In Study 1b, a new sample of participants evaluated these items numerically for how typical they believed each item to be in the context of a hate crime. Study 2 then provided participants, in an experimental format, with written case summaries that either did or did not contain various prototype elements to determine their impact on legal decision making. Both studies received ethics approval from the York University’s research ethics board.

## Study 1a

### Participants

Participants were recruited from a blended sample of community members and university undergraduate participants. Roughly half of participants (*n* = 118) were recruited from introductory psychology classes at a large Canadian university in exchange for partial course credit. The second wave of participants (*n* = 102) were recruited from community members registered with a Qualtrics participant panel in exchange for financial compensation. Participants who did not understand the definition of a hate crime or who showed poor English comprehension were excluded from participation (*n* = 19). We adopted a fairly flexible working definition of a hate crime for the data cleaning process, accepting general descriptions of an offense that is motivated by bias toward the victim’s social group. Most participants did have a clear understanding of this concept. The final sample consisted of 201 participants (123 women, 77 men) with an average age of 31.42 years, ranging from 18 to 82 years. Table 1 presents the demographic characteristics of this sample.

**Table 1.** Demographic Characteristics of Participants.

	Study 1a		Study 1b		Study 2	
Particiant Identity	*N*	%	*N*	%	*N*	%
Ethnicity						
White	89	44.28	60	20.69	75	25.34
Black	27	13.43	34	11.72	17	5.74
East Asian	27	13.43	43	14.83	39	13.18
South Asian	26	12.94	74	25.52	86	29.05
Middle Eastern	13	6.47	42	14.48	33	11.15
Latino/Latina	9	4.48	14	4.83	8	2.70
Other/multiethnic	10	4.98	7	2.41	36	12.16
Declined answer	0	0.00	16	5.52	2	0.68
Religion						
Atheist/Agnostic	41	20.40	48	16.55	51	17.23
Christian/Catholic	90	44.78	107	36.90	100	33.78
Jewish	7	3.48	7	2.41	8	2.70
Muslim	25	12.44	54	18.62	61	20.61
Hindu	5	2.49	28	9.66	21	7.09
Sikh	4	1.99	16	5.52	22	7.43
Buddhist	6	2.99	5	1.72	11	3.72
Other/multifaith	23	11.44	22	7.59	19	6.42
Declined answer	0	0.00	3	1.03	3	1.01

### Materials and Procedure

Following traditional prototype methods (Louden & Skeem, 2007; Smith, 1991, 1993), participants were informed that we were interested in their thoughts and understanding of a hate crime and instructed them to take a few minutes to think about what a typical hate crime might look like. This has been shown to facilitate prototype-relevant information (Skeem & Golding, 2001). Participants were then given a series of open-ended questions probing their thoughts about a typical hate crime victim, hate crime perpetrator, and hate crime offense. This open-ended task allowed participants to report as much detail as they felt appropriate. There were few, and only minor, differences between the community and student samples, and the results are presented collapsing the two samples.

## Results

Open-ended responses were read in their entirety, and common concepts were identified. A coding guideline was prepared for each of the victim, perpetrator, and offense characteristics. Two trained coders then assessed the open-ended responses for the presence or absence of these themes, and any disagreement between the two coders was resolved by mutual agreement. A final count of frequencies of the various themes appears in Table 2, alongside their inter-rater reliabilities. Following the paradigm employed by Smith et al. (2007), items that were identified by at least 10% of the sample are presented as potential prototype content. We present items that showed good internal consistency among the coders (i.e., κs of .60 or higher, conventionally accepted as good inter-rater reliability: e.g., McHugh, 2012).

**Table 2.** Prototype Traits Identified by At Least 10% of Participants in Study 1a and Their Numeric Rating in Study 1b.

	Study 1a			Study 1b					
Prototype Trait	*N*	%	κ	*M*	*SD*	*t*	*df*	*p* ^a^	*d* ^b^
Victim traits									
Racial minority	109	54.50	.688	4.28	0.97	22.48	289	.001	1.32 [1.16, 1.48]
Religious minority	102	51.00	.779	4.26	0.97	22.07	289	.001	1.30 [1.16, 1.47]
Gay/lesbian/bisexual	60	30.00	.880	4.14	1.06	18.27	286	.001	1.08 [0.93, 1.22]
It could be anyone	50	25.00	.828	4.08	1.11	16.61	289	.001	0.98 [0.84, 1.12]
Polite/mild-mannered	32	16.00	.755	3.51	1.02	8.59	287	.001	0.51 [0.38, 0.63]
Minding own business	28	14.00	.667	3.81	1.03	13.36	287	.001	0.78 [0.65, 0.92]
Gender minority	26	13.00	.651	4.03	1.12	15.57	287	.001	0.92 [0.78, 1.05]
Perpetrator traits									
Prejudice/ignorance	101	50.50	.611	4.29	0.93	13.61	288	.001	1.39 [1.23, 1.55]
White perpetrator	67	33.50	.824	3.89	1.00	15.38	288	.001	0.90 [0.76, 1.03]
Male perpetrator	43	21.50	.811	3.99	0.93	18.07	288	.001	1.06 [0.92, 1.21]
Organized hate group	36	18.00	.816	3.68	1.07	10.82	288	.002	0.64 [0.51, 0.76]
Low status	34	17.00	.771	3.13	1.07	2.03	288	.022	1.20 [0.01, 0.24]
Offense traits									
Physical violence	110	55.00	.741	4.07	0.92	19.61	285	.001	1.16 [1.01, 1.31]
Racist motivation	109	54.50	.677	4.40	0.87	17.36	288	.001	1.61 [1.43, 1.78]
Religious bias	74	37.00	.642	4.35	0.92	24.99	288	.001	1.47 [1.30, 1.64]
Verbal harassment/slurs	67	33.50	.743	4.37	0.89	26.00	286	.001	1.54 [1.36, 1.71]
Property damage	54	27.00	.830	3.54	1.10	8.33	287	.261	0.49 [0.37, 0.61]
Homophobic motivation	40	20.00	.751	4.16	1.06	18.62	287	.001	1.10 [0.95, 1.24]
Symbolic location	36	18.00	.808	3.90	1.03	14.69	286	.001	0.87 [0.73, 1.00]
Any time or place	28	14.00	.751	4.33	0.93	24.34	287	.001	1.43 [1.27, 1.60]
Islamophobic motivation	28	14.00	.838	4.21	1.03	19.94	287	.001	1.17 [1.02, 1.32]
Anti-Black bias	26	13.00	.954	4.20	1.05	19.41	288	.001	1.14 [0.99, 1.29]
Occurs at night	24	12.00	.825	3.75	0.94	13.50	286	.001	0.80 [0.66, 0.93]

*Note*. CI = confidence interval; *SD* = standard deviation.

a *t* tests reported test against the midpoint scale rating, with a one-side *p*-value reported.

b Data in brackets corresponds to 95% CIs for Cohen’s *d* values.

### Prototype Content

With respect to the typical hate crime victim, the most common traits pertained to the group identity of the victim, most often a victim who is a racial minority (54.50%), a religious minority (51.00%), a sexual orientation minority (i.e., gay, lesbian, or bisexual, 30.00%), or a gender minority (i.e., non-binary or transgender, 13.00%). Interestingly, many participants also pointed out that the victim of a hate crime could be anyone (25.00%) and that the victim would be someone who was innocent and minding their own business (e.g., “a nice person in the wrong place at the wrong time,” “innocent and unsuspecting,” “someone just walking down the street”; 14.00%), or someone who was mild-mannered and passive (e.g., “polite, sweet, kind, vulnerable, quiet, shy”; 16.00%). Regarding prototypes of a hate crime perpetrator, participants most often reported that they would be overtly biased, prejudiced, and bigoted (50.50%), White (33.50%), and male (21.50%). Some participants suggested that the perpetrator might have ties to organized hate groups (e.g., “extremist groups like the KKK,” “White supremacist group”; 18.00%) and that the perpetrator would be someone of lower socioeconomic status (e.g., low education and wealth; 17.00%).

The offense itself was most often thought to involve an act of interpersonal violence (55.00%), motivated by racial bias (54.50%), religious bias (37.00%), homophobia (20.00%), Islamophobia (14.00%), or anti-Black racism (13.00%). Participants also believed that the offense would be marked by the presence of verbal harassment or slurs (e.g., “derogatory comments,” “racial slurs,”; 33.50%) or property damage (e.g., “graffiti on a synagogue or mosque,” “vandalism,” “rude words written on a home”; 27.00%). Some participants reported that a hate crime could happen at any time or place (14.00%), and others believed that it might be committed in a place of symbolic importance to the victim’s group (e.g., place of worship, community center, and cemetery; 18.00%), and might be more likely to happen at night (12.00%).

It should be noted that other concepts and ideas were generated by participants in lower frequencies. For example, five participants mentioned a disabled victim, three mentioned a White victim being attacked by a racial minority perpetrator, three participants thought the victim would be wealthy or high social status, eight participants mentioned some form of cyber harassment, and three participants thought the perpetrator would escape punishment. These items were not included as part of the central prototype due to their rarity.

## Study 1a—Discussion

The open-ended responses revealed several themes. Hate crimes were thought to involve (a) a victim who is a racial, religious, gender, or sexual orientation minority who is relatively passive and does not provoke the offense; (b) a clearly prejudiced White male who is relatively uneducated but affiliated with organized hate groups, and (c) an act of violence involving verbal harassment/slurs, or property damage to a symbolic location. To determine whether these elements were indeed representative of hate crime prototypes, a new sample of participants was recruited to numerically rate each element identified in Study 1a for how characteristic it was of a hate crime.

## Study 1b—Method

### Participants

Participants were recruited from undergraduate psychology classes and received partial course credit for their participation. Six participants were excluded from analyses due to inattentive responding (i.e., all responses containing an identical numeric response), leaving a final sample of 290 participants, comprising 169 women, 116 men, 3 who identified with a non-binary gender, and 2 participants who declined to answer (*M*_age_ = 19.90 years, standard deviation [*SD*] = 3.41, ranging 17–40 years) (see Table 1 for demographic characteristics).

### Materials and Procedure

Participants were asked to “think for a few minutes about what you know about hate crimes and what images come to mind. Form an image or story in your mind about what kind of a crime has occurred, who was victimized, and who might have committed the offense.” They were then presented with a list of the traits identified in Study 1a and asked to rate each for how typical it was of a hate crime on a Likert-type scale (1—*Not at all Typical* to 5—*Extremely Typical*). After completing the rating task, participants were presented with a list of the items generated in Study 1a and asked to select up to 5 items from this list that they thought were most important to their concept of a hate crime. In this selection task, participants were able to click and drag up to 5 items from the list into the prototypical column. This ranking task was used to identify the more common themes across the victim, perpetrator, and offense characteristics. To reduce redundancy, we collapsed together racial, religious, and sexual orientation minorities into one conceptually related trait to allow for additional prototype elements to be selected for the “top five” list (e.g., rather than having identity-based items fill the list when they tap into the same essential element).

## Results

### Typicality Ratings

Table 2 presents the mean typicality scores for all items, organized by victim, perpetrator, and offense traits. Higher scores indicate that an item is believed to be more typical of a hate crime. Each mean was tested against the midpoint of the scale (i.e., 3.00, neither atypical nor typical) with a one-sided test to determine whether each item was significantly more typical of a hate crime. Collapsing across victim, perpetrator, and offense categories, several elements emerged as most prototypical of a hate crime. A racial minority victim, a racially-motivated offense, and anti-Black motivation were all rated as high in typicality, as were a religious minority victim, religious hate motivation, and Islamophobic hate motivation. Similarly, a gay/lesbian/bisexual victim and a homophobic/biphobic motivation received high typicality ratings. These we may consolidate into related prototypes of hate as being motivated by racism, religious bias, or homophobia. Also prototypic was a perpetrator who is an overtly prejudiced White male, low in socioeconomic status, with some affiliation to organized hate groups. A prototypic offense was characterized by an act of interpersonal violence, accompanied by slurs and verbal harassment, and a victim who is polite/passive and who was minding their own business at the time of the offense.

### Ranking Task

As a second validation of prototype content, participants were directed to a selection task, in which they selected up to 5 items that they believed were most typical of a hate crime and rank ordered them in terms of typicality. The most commonly selected item was a victim who is a racial, religious, or sexual minority, with 80.69% of participants selecting this item as highly typical of a hate crime. A large majority (71.38%) selected offensive slurs and verbal harassment as highly prototypical, and 65.17% believed that the victim would be an innocent person minding their own business at the time of the offense. More than half of the sample (56.90%) believed that a typical hate crime would involve violence, 43.45% believed it would occur in a public place, and 40.34% believed the typical perpetrator would be White.

## Discussion

The results of Studies 1a and 1b provide a clear picture of the perceived nature and content of hate crime prototypes. Prototypical hate crimes are thought to involve a racial, religious, or sexual minority victim, harassment and slurs, a victim minding their own business, and a White male perpetrator. One factor influencing how observers interpret hate crimes may involve the way in which responsibility and blame are apportioned between the victim and perpetrator. Unfortunately, victims of all crimes may be deemed responsible for their own victimization, particularly where people believe in a just world in which people get only what they deserve (Lerner, 1980). An emerging literature has looked specifically at victim blaming in the context of hate crimes and has found that the presence of explicit hate motivation reduces victim blaming and increases perpetrator blame (e.g., Rayburn et al., 2003; Saucier et al., 2010).

Novel to the present research, however, is the finding that hate crime victims are considered to be relatively innocent and minding their own business. Little experimental research on hate crimes has depicted the victim as anything other than passive or a mere placeholder for an identity. For example, Rayburn et al. (2003) depicted the victim as quietly walking home alone when attacked by two males shouting offensive slurs. Saucier et al. (2006) depicted interactions in which a perpetrator assaulted the victim and shouted slurs after only a brief verbal exchange. In another study, the victim merely said “hi” to the perpetrator in a bar and walked away when he was followed and beaten (Plumm et al., 2015). The few studies that have humanized hate crime victims by describing their behavior show an increase in victim blame, such as when a Black victim reacted verbally to a racist comment (Lyons, 2006), a man asked another man to dance (Plumm et al., 2010) or gay or Indigenous victim was observed marching in a parade (Plumm et al., 2014). Erentzen et al. (2021) found that South Asian Muslim victims who responded to harassment were more likely to be blamed for a subsequent assault. If there are behavioral expectations of how a victim should behave during a hate crime, this may lead to discrepant outcomes for victims who do not conform to these expectations. It is unclear, however, how elements high in prototypicality might influence legal decision making and blame attributions pertaining to hate crime; thus, a second study was designed.

## Study 2

Study 2 explored how the presence or absence of prototype elements might influence legal decision making about bias-motivated offenses. Within the study, the perpetrator and offense were held constant as more prototypic, with a White male perpetrator who utters offensive harassment during an interaction. The situation depicted was intentionally ambiguous, such that participants could interpret the events as depicting a hate crime, an unpleasant disagreement that escalated to violence, or an act of self-defense. Participants could disambiguate the situation as they thought it most intuitive. Victim prototype content, however, was varied with respect to two key elements. First, we varied the racial identity of the victim as either Black (prototypic) or White (non-prototypic). Second, following earlier work (Erentzen et al., 2021), we varied the victim’s behavioral reaction to the offender’s verbal harassment prior to the assault, such that the victim either passively ignored the harassment (prototypic), verbally responded to it (less prototypic) or became verbally and physically confrontational (non-prototypic). Study 2 assessed not only the degree to which an offense was thought to be a hate crime but also the extent to which the victim and perpetrator were blamed for the offense. It was hypothesized that when the victim was described as Black as opposed to White (i.e., more prototypic), participants would be more likely to interpret the offense as a hate crime, show higher perpetrator blame, and lower victim blame. It was further hypothesized that when the victim was depicted as passive (i.e., more prototypic), participants would be more certain the offense was a hate crime, with higher perpetrator blame and lower victim blame. The potential interaction between victim race and victim reaction was explored, with the belief that the presence of additional prototype elements would lead to the greatest recognition of the offense as hate motivated, resulting in the highest perpetrator blame and lowest victim blame.

## Method

### Participants

Participants were recruited from introductory psychology classes at a large Canadian university in exchange for partial course credit. Participants were excluded if they failed one or more attention checks (*n* = 36) or if they failed a manipulation check (*n* = 80). This resulted in a final sample of 296 participants (131 men, 160 women, 4 declining to answer). The mean age of the sample was 20.48 years (*SD* = 3.97), ranging from 17 to 50 years (see Table 1 for demographic information).

### Materials

#### Vignettes

Participants were presented with one of six written case summaries depicting a scenario in which a young man (either White or Black) was taking part in a public demonstration to raise awareness about issues facing their community. A White man walking by the demonstration verbally harasses the protester, saying that the city would be better without people like him in it. The protesters then responded in one of three ways. In the non-reactive/passive victim condition, he simply ignores the comments and looks away. In the verbal reaction condition, the victim says, “What did you just say to me?” but otherwise remains where he is. In the physical reaction condition, the victim says, “What did you just say to me?” and then aggressively walks toward the man and shoves him backward. The perpetrator then escalates the incident and physically assaults the victim while shouting offensive comments.

#### Certainty of Hate

Participants reported whether they believed the offense was a hate crime (yes or no) and rated their certainty in this decision (1—*Not at all Certain* to 7—*Completely Certain*). Those who indicated that the offense was a hate crime were given a score of +1, and those who responded that the offense was not a hate crime were given a score of −1. This was then multiplied by their confidence rating in this decision, with their product constituting a measure of participants’ certainty that the offense was a hate crime. This measure ranged from −7 (*completely certain this was not a hate crime*) to +7 (*completely certain this was a hate crime*).

#### Blame Ratings

Evaluations of perpetrator blame were made on a series of items that involved both affective and cognitive items. On a 7-point scale (1—*Not at All*, 7—*Completely*), participants reported the degree to which the perpetrator made them feel anger, sympathy (reverse coded), positive emotions (reverse coded), the degree to which the perpetrator acted reasonably (reverse coded) and was responsible for the events. These items were summed and averaged into a composite measure of perpetrator blame (α = .77), with similar evaluations rendered for victim blame (α = .82).

### Procedure

After providing informed consent, participants were randomly assigned to one of the six conditions in this 2 (victim race: Black vs. White) × 3 (victim reaction: non-reactive, verbal, physical) between-subjects factorial design. After reading the case transcript, participants completed manipulation checks, evaluations of the case as a hate crime, perpetrator blame, victim blame, and demographics. Upon completion, participants were debriefed and thanked.

## Results

The data were analyzed with a series of 2 (victim race) × 3 (victim reaction) univariate analyses of variance across the dependent variables (i.e., certainty of hate, perpetrator blame, and victim blame). Post hoc comparisons were made using a Bonferroni error correction.

### Hate Crime Certainty

**Figure 1.**
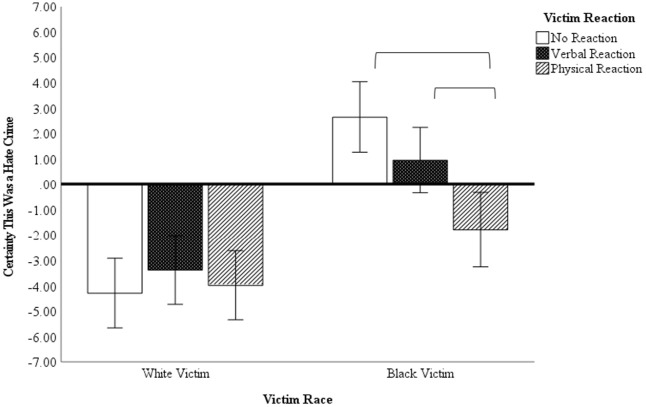
Certainly, the offense is a hate crime as a function of victim race and victim behavior.

There was a significant main effect of victim race, *F* (1, 290) = 62.05, *p* < .001, η^2^ = .176, and victim reaction, *F* (1, 290) = 4.77, *p* = .009, η^2^ = .032. These main effects were qualified by a significant interaction between these two variables, *F* (2, 290) = 5.62, *p* = .004, η^2^ = .037. As seen in Figure 1, for the White victim conditions, there were no differences in certainty of hate as a function of victim behavior, with participants evaluating the offense equivalently across the three victim reaction categories (all *p*s > .357). In other words, participants did not view the offense as a hate crime when the victim was White, regardless of the victim’s behavior. In the Black victim conditions, however, the non-reactive and physically reactive victim conditions differed significantly (*p* < .001), as did the verbal and physical victim reaction conditions (*p* = .006). This suggests a degree of behavioral scrutiny for Black victims that does not occur for White victims. As an alternative approach, we may compare victim race within each behavioral condition. Unsurprisingly, participants were more certain the offense was a hate crime when it targeted a Black victim compared to a White victim in the passive (*p* < .001), verbally responsive (*p* < .001), and physically responsive conditions (*p* = .032).

### Perpetrator Blame

**Figure 2.**
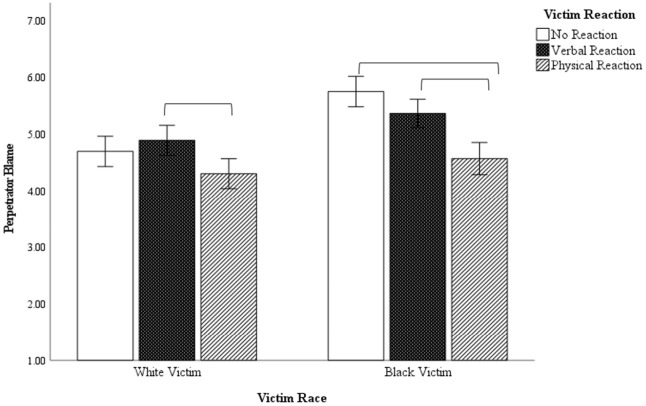
Perpetrator blame ratings as a function of victim race and victim behavior.

The ANOVA examining perpetrator blame ratings revealed a main effect of victim race, *F* (1, 290) = 29.22, *p* < .001, η^2^ = .092, and a main effect of victim reaction, *F* (1, 290) = 19.44, *p* < .001, η^2^ = .118. As seen in Figure 2, these main effects were qualified by an interaction of victim race and victim behavior, *F* (2, 290) = 4.44, *p* = .013, η^2^ = .030. Within the White victim conditions, perpetrator blame was equivalent in the non-reactive and verbal reaction conditions (*p* = .920) and the non-reactive and physical reaction conditions (*p* = .120). Although perpetrator blame was lower in the physical reaction condition, this difference was significant only for the comparison between the verbal and physical reaction conditions (*p* = .006). In the Black victim condition, perpetrator blame was lower when the victim was physically versus verbally responsive (*p* < .001), and when the victim was physically reactive versus non-reactive (*p* < .001), but the no reaction versus verbal reaction comparisons did not reach significance (*p* = .119). Again, perpetrator blame showed less variation in relation to the White victim’s behavior compared to the Black victim’s behavior. As an alternative comparison, we may look at victim race within each behavioral condition. Perpetrators were blamed more for attacking a Black victim than a White victim in the passive (*p* < .001) and verbally responsive (*p* < .011) conditions, but there was no difference by victim race in the physically confrontational conditions (*p* = .178).

### Victim Blame

**Figure 3.**
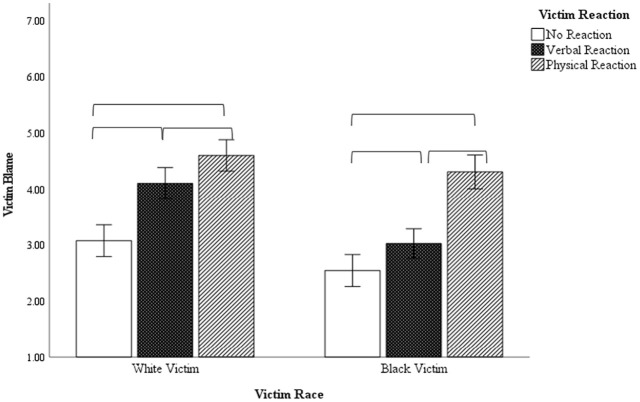
Victim blame ratings as a function of victim race and victim behavior.

The ANOVA examining victim blaming revealed a significant main effect of victim race, *F* (1, 290) = 29.50, *p* < .001, η^2^ = .092, and victim reaction, *F* (1, 290) = 63.61, *p* < .001, η^2^ = .305. These were again qualified by a significant interaction, *F* (1, 290) = 4.06, *p* = .018, η^2^ = .027. As seen in Figure 3, within the White victim condition, victim blaming was lowest when the victim was non-reactive, increased significantly as the White victim became verbally responsive (*p* < .001), and approached conventional significance thresholds as the White victim became physically responsive (*p* = .042). In the Black victim conditions, victim blaming was lowest in the non-reactive victim condition compared to the verbal condition (*p* = .047) and the physically reactive condition (*p* < .001). An alternative way to assess these results is to compare victim race within each behavioral condition. Black victims received less blame than did White victims in the passive (*p* = .009) and verbally responsive conditions (*p* < .001), but there was no longer a difference by victim race in the physically confrontational condition.

## Study 2—Discussion

These results reveal that determinations of a hate crime are strongly influenced by victim identity, such that offenses involving a White victim were not considered hate crimes regardless of the White victim’s behavior. Offenses involving Black victims were more likely to be considered a hate crime, but this was more affected by the Black victim’s behavior. When the Black victim was passive, participants typically believed the offense was hate-motivated, but this decreased as the Black victim became verbally and physically confrontational. In addition, victim blame and perpetrator blame were influenced by the victim’s behavior, but these effects were stronger for Black victims than for White victims. These findings align with previous research in which hate crimes that fit the stereotypical pattern of a violent assault by a White male against a minority male are more likely to receive higher guilt ratings (Craig & Waldo, 1996; Erentzen et al., 2021; Lyons, 2006; Saucier et al., 2006). Prior research has found that participants tend to ascribe a sort of “victim halo” to victims of hate crime, wherein we see reduced victim blaming, higher victim sympathy, and more favorable emotional reactions to hate crime victims than to non-hate crime victims. It appears that a similar process may occur here as well, in that crimes that fit a prototype of an innocent racial minority attract sympathy, but as the victim becomes more assertive (hence less prototypical), the hate crime victim halo dissipates.

## General Discussion

The present research has identified the nature and content of hate crime prototypes and their potential consequences for legal decision making. Consistent with earlier work by Smith (1991, 1993), participants were more likely to assign guilt and blame in cases that match their prototype of an offense, even if prototypic elements are not legally relevant. Relying on these prototypes may result in legally inaccurate and even biased judgments (Skeem & Golding, 2001). These findings are consistent with prior research that has found that observers are more likely to interpret an offense as hate-motivated where it involves a victim who is a racial, religious, or sexual orientation minority assaulted by a White male (e.g., Cramer et al., 2013; Lyons & Roberts, 2014; Plumm et al., 2015; Saucier et al., 2006, 2010). It is also consistent with research finding that victims who speak or engage with a perpetrator receive less sympathy, and the offense is less likely recognized as a hate crime (Lyons, 2006; Plumm et al., 2010).

This work is consistent with prior theorizing on ideal victims (e.g., Christie, 2018 [1986]), providing a rare systemic study of both the content and consequences of such prototypes. Christie’s conceptualization of a weak victim engaging in respectable actions is reflected in the prototypes identified herein of a passive and non-reactive victim; the conceptualization of an offender as “big and bad” is seen in the prototypic expectation that the offender is a lower status, ignorant person with clear expressions of antagonism. Unique to a hate crime prototype is a degree of asymmetry between the offender and victim identities, with the offender having more relative social privilege. The expectation of a passive racialized minority victim (but not a passive White victim) suggests that prototype content works in a complex manner, not merely additive but mutually determinative.

### Practical Implications

One troubling finding from the present research is that Black victims of hate crime in North America may be subjected to legally extraneous considerations, particularly the expectation that a “true” hate crime victim is one that remains passive in their response to harassment. Prototypes of hate crime may influence how witnesses, victims, and those close to victims interpret the offense as hate-motivated. When less prototypical, there may be a reduction in willingness to report the offense to police or a belief that it will not be taken seriously. It is important to understand how such prototypes may affect recognition of offenses as hate-motivated, willingness to report to police, and eventual case handling by criminal justice professionals.

Only 15 to 20% of hate crimes are reported to police in North America (e.g., Pezzella et al., 2019), and fewer still are investigated or prosecuted. Hate crime prototypes can influence whether a victim feels comfortable reporting their experiences to their own social network; prototypes may also affect how those social networks of friends and family respond to that victim, which can, in turn, affect the decision to report and access support services and compensation. Further, police may be affected by subconscious prototypes of what a “real” hate crime looks like, ultimately affecting the decision to investigate, lay charges, and record an offense as hate motivated, all of which affects statistical recording of hate crime and allocation of resources to affected communities (Wiedlitzka et al., 2018). A hate crime that involves a victim who is vocal or agentic, who is not a racial or other minority, a perpetrator who is not “big and bad,” or where there are other non-prototypic elements may be less likely to be handled as a hate-based offense.

Although the present work is preliminary, it suggests the potential need for enhanced police training about hate crimes and the risks of relying on prototypic representations. At present, there is little training for police on hate crimes, inconsistent definitions of what a hate crime is, and a lack of a dedicated hate crimes unit in most police forces (Perry & Samuels-Wortley, 2021). Enhanced training that includes warning against reliance on prototypes or victim passivity would be warranted, as well as the potential use of warnings to juries in a trial context.

The notion that offenders must be “big and bad” (Christie, 2018 [1986]) and objectively low status and biased can create problematic expectations. As Perry (2001) notes, many hate crime perpetrators believe themselves to be the defenders of public norms and decency, often viewing their actions as morally justified. This may obscure recognition of hate motivation, particularly where the observer shares the offender’s sentiments (e.g., Godzisz & Mazurczak, 2023). Relatedly, one important consideration is the role of the observer’s personal beliefs and attitudes toward the victim’s group identity. One the one hand, it is possible that those with prejudice toward that group will be less likely to recognize an offense as hate motivated, more willing to overlook or minimize hate motivation. On the other hand, those with sympathy or positive sentiment toward the victim’s group may be more likely to recognize an offense as hate motivated, even if hate motivation is unclear or absent. The present research is not able to speak to these possibilities, and additional research is warranted to assess these questions in a directed and considered manner. Future work may also wish to explore the role of intersectionality of the victim’s identity, as prior research has shown that victims experience hate crime as targeting them on the basis of race, gender, age, sexual orientation, and other identities altogether rather than targeting one identity in isolation (Erentzen & Schuller, 2020). How might recognition and interpretation of hate crime be affected by the multiple, overlapping identities of the victim? Would passivity expectations apply equivalently to Black men and Black women?

### Limitations and Future Directions

As with all research, the present work had several limitations. Student samples have been found to differ from the general population with respect to various individual differences (e.g., lower punitiveness, lower authoritarianism, and better reading comprehension; Weiner et al., 2011). On this note, however, prototype content was elicited from both student and community samples, showing few differences between the samples. Both the student and community samples were diverse with respect to ethnicity, religion, and age, offsetting concerns about homogenous participant samples. A second potential limitation involves the use of a written case summary, which may lack external validity. The written summary, however, was chosen as a preferred first step in this paradigm for its superior experimental control. We note that other authors have advocated the use of experimental vignettes as enhancing internal validity without compromising external relevance (e.g., Aguinis et al., 2014). Future research may extend these research questions with longer transcripts, video-recorded mock trials, or court simulations for enhanced external validity. It is also recommended that future directions consider hate crimes that target traditionally non-prototypical and understudied targets of hate (e.g., victims targeted on the basis of disability, political beliefs, mental illness, or subculture).

A final concern may involve the scenario employed in Study 2, in which the victim was described as holding a demonstration to raise awareness about issues affecting his community. This backdrop might have sensitized participants to the concept of inequality and prejudice, enhancing sympathy for the racialized victim more so than for the White victim. An alternative scenario might explore these effects in a different context; as explained in the endnote, we tested these effects with an alternative paradigm (e.g., the victim is thought to be either dealing drugs or leaving a volunteer shift) and observed similar findings.^1^ The ambiguity of the situation may have left too much unclear for participants to understand (e.g., perhaps this was a case of self-defense). We note that this ambiguity does not explain or undermine the pattern of results observed - the victim’s behavior was scrutinized only when he was described as a racial minority. Future studies may wish to consider alternative scenarios that incorporate less ambiguity or interaction between the victim and perpetrator. We also note the possibility that prototypes may be influenced by context, culture, history, and personal experience. We provide this work as a first step toward understanding the nature and content of hate crime prototypes at a general level and as a springboard for further understanding and study.

## Concluding Comments

To the degree that prototypes of hate crimes affect legal outcomes such as police behavior or judicial responses, reactions of friends and family, and the victim’s willingness to report their experience to police, it is critical to understand how these prototypes operate and perhaps how to counteract their pernicious influence. The present research suggests that observers may have subtle expectations of a model hate crime victim: a minority group member who is polite, passive, and accepts harassment with good grace. If this is indeed the case, it suggests an unsettling reality for victims of hate. The existence of hate crime is a violation of democratic principles of equality, multiculturalism, and human dignity. Our understanding of the potential expectations placed on victims to conform to prototypic expectations is an important step toward combatting the harms of hate crime.

## Notes

1. To address this concern, we conducted a follow-up study with a modified scenario. We focused in particular on the non-reactive victim condition, comparing how White vs. Black victims might be evaluated when we remove the inequality prime. Participants (*n* = 210) were recruited from the same participant pool as in Study 2 and randomly assigned to read one of two vignettes describing an assault in a public park. In this scenario, the victim (either Black or White) is in the park after volunteering at a homeless shelter, exchanging phone numbers with fellow volunteers. The perpetrator mistakenly believes he is witnessing a drug deal and confronts the victim while shouting offensive slurs against his race (the victim and perpetrator always differ in racial identity). The victim ignores this harassment and is then violently assaulted. Participants completed the same measures of victim blame (α = .81), perpetrator blame (α = .81), and certainty of hate items presented in Study 2. Crimes targeting a passive Black victim were more likely to be interpreted as a hate crime (*M* = 3.87, *SD* = 3.89) compared to crimes targeting a passive White victim, *M* = −0.29, *SD* = 4.88, *t* (207) = −6.77, *p* < .001, *d* = −0.95. Moreover, crimes involving a passive Black victim attracted more perpetrator blame (*M* = 5.83, *SD* = 1.00) than did offenses involving a passive White victim, *M* = 4.72, *SD* = 1.07; *t* (208) = 7.84, *p* < .001, *d* = −1.08. Offenses involving a passive Black victim attracted less victim blame (*M* = 2.26, *SD* = 0.99) than did crimes involving a passive White victim, *M* = 3.30, *SD* = 1.16, *t* (208) = 7.05, *p* < .001, *d* = 0.97. Together, these results confirm the pattern of results obtained in Study 2, even when the potential inequality prime is removed.
